# Reduction in Pain and Pain Intensity with Nonpharmacological Treatment in Severely Obese Patients: A Randomized Clinical Trial

**DOI:** 10.3390/ijerph182111112

**Published:** 2021-10-22

**Authors:** Carolina Rodrigues Mendonça, Matias Noll, Camila Kellen de Souza Cardoso, Annelisa Silva Alves de Carvalho Santos, Ana Paula dos Santos Rodrigues, Erika Aparecida Silveira

**Affiliations:** 1 Programa de Pós-Graduação em Ciências da Saúde, Faculdade de Medicina, Universidade Federal de Goiás, Goiânia 74650-050, GO, Brazil; matias.noll@ifgoiano.edu.br (M.N.); camilacardoso_nut@hotmail.com (C.K.d.S.C.); annelisa.nut@gmail.com (A.S.A.d.C.S.); anapsr@gmail.com (A.P.d.S.R.); erikasil@terra.com.br (E.A.S.); 2 Instituto Federal Goiano, Rodovia Go-154, Km 03, s/n, Ceres 76300-000, GO, Brazil; 3 Department of Sports Science and Clinical Biomechanics, University of Southern Denmark, 5230 Odense, Denmark

**Keywords:** musculoskeletal pain, chronic pain, morbid obesity, diet, olive oil

## Abstract

The objective of this study was to analyze the effectiveness of two nonpharmacological interventions—traditional Brazilian diet (DieTBra), and extra-virgin olive oil (EVOO)—in terms of the reduction in pain and pain intensity in individuals with severe obesity. We conducted a 12-week parallel randomized clinical trial with 149 individuals (body mass index (BMI) ≥ 35 kg/m^2^) who were randomized into three groups: supplementation with EVOO (*n* = 50), DieTBra (*n* = 49), and EVOO + DieTBra (*n* = 50). Of the total, 133 individuals with a mean BMI of 46.04 kg/m^2^ completed the study. By the end of the follow-up, there was a reduction in severe pain in the EVOO + DieTBra group (*p* = 0.003). There was a significant reduction in severe pain in the EVOO + DieTBra group (−22.7%); pain in the wrist and hand (−14.1%), upper back (−26.9%), and knees (−18.4%) in the DieTBra group; and reduction in hip pain (−11.1%) with EVOO consumption. We conclude that EVOO and DieTBra, either alone or in combination, are effective interventions to reduce pain intensity and pain in different regions in individuals with severe obesity, and have great potential for clinical application.

## 1. Introduction

Obesity and severe obesity (BMI ≥ 35 kg/m^2^) have increased alarmingly worldwide, and are risk factors for many diseases, including musculoskeletal disorders [1,2]. In adults with obesity, musculoskeletal disorders are the second leading cause of disability [1]. In addition, obesity is associated with increased musculoskeletal pain (pain arising from structures such as muscles, bones, or joints) [3,4,5,6,7], and frequently reported as multisite pain—that is, pain that occurs simultaneously at more than one anatomical site [4,5,8]. Thus, considering the relationships between pain and obesity, and the presence of multimorbidity, it is relevant to investigate the possibilities of treatment for musculoskeletal pain in individuals with severe obesity—primarily nondrug treatments with low costs and no side effects [1,4].

In this context, the daily consumption of certain foods—such as fruits, vegetables, yogurt, red wine, and extra-virgin olive oil—can modulate pain [9,10,11]. Many of these food items are included in the traditional Brazilian diet (DieTBra), such as rice, beans, fruits, and vegetables [12]. However, there are no previous studies evaluating the effects of DieTBra on musculoskeletal pain. The few nutritional interventions to have investigated the treatment of pain were performed on adults with osteoarthritis and rheumatoid arthritis (RA) [13], to investigate specific foods that affect RA symptoms [13], testing the Mediterranean diet [14], a gluten-free diet, and a vegan diet [15,16]. However, the long-term effects of these interventions require further study [14].

Extra-virgin olive oil (EVOO) contains phenolic compounds and monounsaturated fatty acids [17]. Research on the effects of EVOO on pain management has been performed only by supplementation with capsules [18], or topical application of gel or ointment [19,20]. There are also some studies on the effects of the Mediterranean diet, which has olive oil as a primary source of added fat, on pain [14,21]. However, the effect of EVOO as an isolated food, or in the context of another food pattern other than the Mediterranean diet, has not yet been evaluated. We found no evidence of the effects of EVOO consumption as food on pain in adults with or without obesity.

Considering the high prevalence of pain, its close relationship with obesity, and the potential benefits of DieTBra and EVOO, we propose the present study. We believe that DieTBra and EVOO may be a pathway for the treatment of pain in individuals with obesity. Thus, the objective of this study was to analyze the effectiveness of two nonpharmacological interventions—the traditional Brazilian diet, and extra-virgin olive oil—in terms of the reduction in pain and pain intensity in individuals with severe obesity.

## 2. Materials and Methods

### 2.1. Study Design and Ethical Aspects

This was a 12-week randomized, controlled, parallel clinical trial with severely obese (BMI ≥ 35 kg/m^2^) adult individuals [22,23,24,25,26]. This study is part of the DieTBra Trial, which is an interdisciplinary study evaluating different outcomes [22,23,24,25,26]. Data collection took place between June 2015 and February 2016, in the Nutrition in Severe Obesity Outpatient Clinic (ANOG) of the Clinical Hospital/ Federal University of Goiás **/**(HC/UFG), in partnership with the Clinical Research Unit of UFG, Goiás State, Brazil.

This research was approved by the Research Ethics Committee (CEP/HC/UFG) under protocol no. 747792. Ethical aspects were respected, in accordance with the Helsinki Declaration. This study is in line with the recommendations of the Consolidated Standards for Reporting Trials (CONSORT), and was registered at ClinicalTrials.gov (NCT02463435). All study participants signed an informed consent form.

### 2.2. Participants and Inclusion and Exclusion Criteria

The inclusion criteria were age between 18 and 65 years, BMI ≥ 35 kg/m^2^, and living in the metropolitan region of Goiânia, capital of the state of Goiás, Brazil. The exclusion criteria were a history of bariatric surgery, weight loss ≥ 8% in the past three months [27], nutritional treatment in the past two years, intolerance to any vegetable oil, pregnancy and lactation, and people with special needs who were not able to walk, hear, or speak. In addition, individuals with HIV/AIDS, chronic obstructive pulmonary disease, cardiac insufficiency, liver or kidney failure, or cancer, as well as those with daily use of anti-inflammatory drugs and corticosteroids, were excluded. Patients with diabetes or on opioid treatment were not excluded from the present study.

### 2.3. Randomization

**Figure 1:**
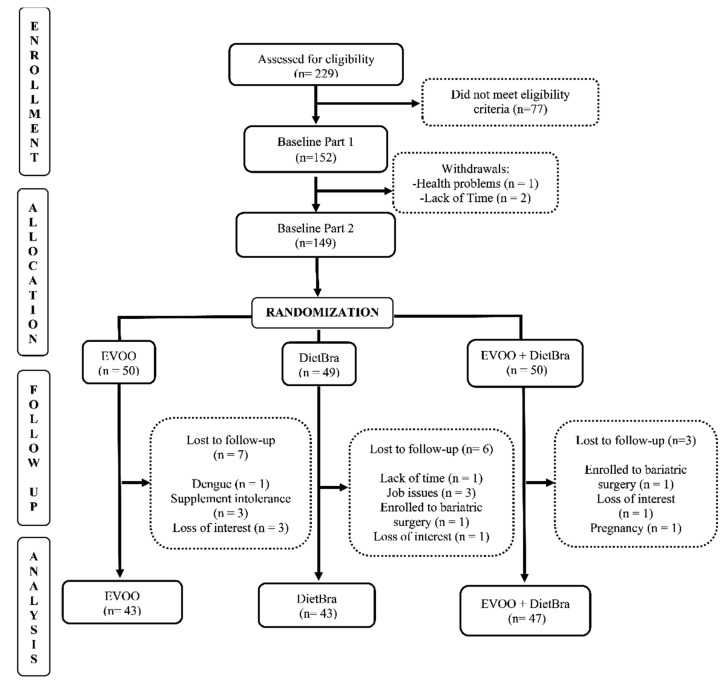
CONSORT diagram for study recruitment.

The individuals included in the study were randomized at baseline into three intervention groups, in the proportion of 1:1:1, using a randomization list generated by the site www.randomization.com (accessed on 1 October 2021). The groups were: (1) extra-virgin olive oil (EVOO); (2) traditional Brazilian diet (DieTBra); and (3) extra-virgin olive oil + DieTBra (EVOO + DieTBra) (Figure 1).

### 2.4. Interventions and Follow-Up

The individuals in the EVOO group were instructed to consume 52 mL of extra-virgin olive oil daily. They received the olive oil in the form of sachets of 13 mL, and were instructed to consume 4 sachets per day, preferably at lunch and/or dinner, at room temperature. This group did not receive any other recommendations, such as nutritional counseling, dietary prescriptions, or recommendations for regular physical activity practice.

In the DieTBra group, after consultation with a registered dietitian, all individuals received an individualized meal plan with a food substitution list, divided into 4–6 meals per day. The food plan was based on DieTBra, where the staple foods during lunch and dinner are rice; beans; a small portion of meat, chicken, or fish; cooked and raw vegetables in the form of salads; and fresh seasonal fruits as dessert. Fruits were also included in the intervals between the main meals [28]. The consumption of other cereals, roots and tubers, milk and dairy products, fish, whole grains, vegetables, and eggs was recommended according to the Food Guide for the Brazilian Population [29].

The food plan took into consideration individualized calculations, aiming at reducing the initial body weight by 5–10%. The daily caloric reduction (550–1100 kcal/day) was determined after defining the weekly weight reduction target (0.5 to 1.0 kg/week), according to the initial weight and BMI of each individual [30]. Total energy values were calculated considering the energy expenditure at rest, using an equation for individuals with severe obesity [31]. The total energy expenditure was found by multiplying the resting energy expenditure by the activity factor, obtained via the Global Questionnaire on Physical Activity and the thermic effect of foods [32]. The Dietary Reference Intake guidelines were followed for the distribution of macronutrients: 45–65% carbohydrates, 10–35% proteins, and 20–35% lipids [33].

The EVOO + DieTBra group received the same intervention as the DieTBra group, along with daily supplementation of 52 mL of EVOO. The supplementation generated a caloric increase of 468 kcal/day, which was discounted from the prescribed food plan to adjust the total energy value.

To evaluate the adherence to olive oil consumption, the individuals were requested to return all consumed and unconsumed sachets in each consultation. To evaluate diet adherence, the 24 h recall and food frequency questionnaires were used. In every consultation, according to each group, the registered dietitians reinforced the importance of using olive oil and following the food plan.

The amount of extra-virgin olive oil supplemented was 52 mL (4 sachets). The product used in this study had <0.2% acidity, and was cold-pressed and packed in photosensitive sachets. The extra-virgin olive oil was obtained from a reputable company, following rigorous quality standards, using funding granted to the larger study. The quality of the product was also tested independently to evaluate ash content, acidity, purity, and the fatty acid profile (data not shown).

The follow-up period was 12 weeks, and consultations with the registered dietitians occurred every 4 weeks.

### 2.5. Blinding

In clinical trials with nutritional intervention, blinding is often difficult and impractical [25,34,35]. However, in this study, patients were blinded to the type of supplement consumed. In addition, some measures were taken to mask the type of intervention, such as consultations on different days for each intervention group in order to avoid contact and exchange of information between the individuals. To ensure blinding, the groups that received the EVOO sachets were informed that they were receiving a food supplement rich in polyphenols, and the terms “olive oil” were never mentioned by any member of the research team. The information on the supplement sachets was adequately prepared according to the recommendations of the National Health Surveillance Agency of Brazil for Clinical Trials to mask this intervention.

### 2.6. Research Team and Quality Control

All team members were trained for data collection. At the end of each consultation, the questionnaires were coded and verified by different team members. To improve patient adherence, a team member was responsible for reminding participants of their consultations by telephone call. In addition, 36 standard operating procedures (SOPs) were developed to achieve uniformity of the procedures and avoid errors.

### 2.7. Demographic and Lifestyle Variables

Demographic (sex and age) and lifestyle variables (smoking and binge drinking) were collected using a previously tested standardized questionnaire. For the smoking variable, the individuals were asked if they smoked or had previously smoked cigarettes/pipes/cigars [36]. Regarding episodes of excessive drinking (binge drinking)—defined as the consumption of five or more doses of any alcoholic beverage on a single occasion for males, and four or more doses for females—this was evaluated via a simplified version (adapted to the present study) of the questionnaire from the Gender, Alcohol, and Culture: an International Study—GENACIS [37,38].

Moderate-to-vigorous physical activity (MVPA) was analyzed using a waterproof ActiGraph wGT3X triaxial accelerometer (ActiGraph, Pensacola, FL, USA). Individuals were instructed to wear the device for six consecutive days, including two weekend days, for 24 h a day, on their non-dominant wrist. The accelerometer frequency was set to 30 Hz, and the data collection interval was set to 1 min. The output data were processed using the GGIR R package [http://cran.r-project.org, accessed on 1 January 2017]. Moderate and vigorous physical activity were assessed in average minutes per weekday spent on activities that lasted at least five consecutive minutes. The variable did not present normal distribution using the Shapiro–Wilk test; therefore, it was categorized by the median.

### 2.8. Anthropometric Variables

Body weight was measured on a platform-type digital scale, with a capacity of 200 kg and precision of 100 g (Welmy, Fishkill, NY, USA). Stature was measured using a stadiometer coupled to a digital scale, with an accuracy of 0.1 cm, and performed according to the Lohman, Roche, and Martorell protocol [39]. Weight and height were used to calculate BMI (BMI = weight (kg)/height (m)^2^). Individuals with obesity were classified as: 35–39.90 kg/m^2^, 40–49.90 kg/m^2^, or >50.00 kg/m^2^ [40].

### 2.9. Clinical Variables

The clinical variables included were prior diagnosis of arthritis or arthrosis, symptoms of anxiety and depression, use of medications, biochemical parameters, and food consumption. The Hospital Anxiety and Depression Scale was applied for the assessment of anxiety and depression symptoms [41]. Medication use was classified according to the Anatomical Therapeutic Chemical Classification System [42], in groups with similar mechanisms of action: analgesics, nonsteroidal anti-inflammatory drugs, and muscle relaxants.

Blood samples for biochemical tests were collected following 12 h of fasting. Semi-quantitative C-reactive protein (CRP) (mg/L) was analyzed by immunochemical agglutination reaction, and classified as reagent when CRP > 6 mg/L, or nonreactive when CRP ≤ 6 mg/L. Vitamin D (ng/mL) was analyzed by electrochemiluminescence, and classified as deficiency when values were below 20 ng/mL, insufficiency for values of 21–29 ng/mL, and sufficiency when above 30 ng/mL [43]. Food consumption variables were daily consumption of fresh fruits and raw, cooked, and/or steamed vegetables, collected using a validated food frequency questionnaire [44].

### 2.10. Musculoskeletal Pain

Musculoskeletal pain in the past seven days was assessed using the Nordic Musculoskeletal Questionnaire (NMQ), adapted and validated to the Brazilian culture and individuals with obesity [45,46]. Outcomes based on pain symptoms were reported for nine anatomical regions: neck, shoulders, elbows, upper back, lower back, wrists/hands, hips/thighs, knees, and ankles/feet [45,47]. Three outcomes were considered using the NMQ: pain (yes and no), pain by body region, and multisite pain [8]. Furthermore, a numerical pain rating scale ranging from 0 to 10 was used to assess pain intensity. Severe pain was classified as ≥8 [48].

### 2.11. Statistical Analysis

The database was structured using EpiDATA^®^ (the EpiData Association, Odense, Denmark) version 3.1, with double-entry typing and data validation. Statistical analyses were performed using Stata version 13.0 (StataCorp LP, College Station, TX, USA) statistical package. Statistical significance was set at 5%.

Descriptive analyses are presented in absolute numbers (*n*) and relative frequencies (%), along with the mean and standard deviation. The outcomes analyzed were presence of pain (yes and no), severe pain, and prevalence of pain in the wrist/hands, upper back, lower back, hips/thighs, knees, and ankles/feet. Pearson’s chi-squared test and McNemar’s test were used to compare proportions. Delta—that is, the difference between the final and the initial moment for each outcome—was calculated using Pearson’s chi-squared test.

## 3. Results

A total of 149 individuals, with a mean age of 39.63 ± 0.72 years and mean BMI of 46.3 ± 0.52 kg/m^2^, participated in the study. The other characteristics of the participants, by intervention group, are described in Table 1.

Mean weight reduction in each group was as follows: DieTBra −2.65 ± 5.54 kg/m^2^, and EVOO + DieTBra −1.64 ± 3.47 kg/m^2^. In the EVOO group, there was a mean weight gain of +1.66 ± 2.94 kg/m^2^. The weight variable did not meet the criteria for ANCOVA analysis, because the assumptions for carrying out the test were not met.

In the analysis comparing the baseline with the end of follow-up in each intervention group, we observed a significant reduction in pain in the upper back (*p* = 0.001) and knees (*p* = 0.046) in the DieTBra group. In the EVOO + DieTBra group, there was a significant reduction in severe pain (*p* = 0.002) (Table 2).

Comparing the three groups at the end of the follow-up, there were no statistically significant differences in the outcome variables; the same occurred when the analysis was performed comparing groups two by two (Supplementary Table S1).

Considering the changes in the prevalence of pain outcomes, several significant differences were detected. For severe pain, EVOO + DieTBra presented greater reduction compared to EVOO alone (*p* = 0.007) and to DieTBra alone (*p* = 0.003). For wrist and hand pain, DieTBra showed greater reduction compared to EVOO + DieTBra (*p* = 0.014). For upper back pain, DieTBra presented the greatest reduction among all intervention groups (*p* = 0.001 compared to EVOO alone; *p* = 0.002 compared to EVOO + DieTBra), and EVOO + DieTBra presented greater reduction compared to EVOO alone (*p* = 0.045). For knee pain, DieTBra presented greater reduction compared to EVOO alone (*p* = 0.028) and EVOO + DieTBra (*p* = 0.016) (Table 3).

## 4. Discussion

This is, to our knowledge, the first study to investigate the effects of EVOO and DieTBra on pain, pain intensity, and pain by site in adults with severe obesity. This study provides evidence that these nutritional interventions, alone or in combination, reduced pain intensity (severe pain) and pain occurrence in different body parts, such as wrists and hands, upper back, and knees. These results confirm our hypotheses of the effectiveness of EVOO and DieTBra on musculoskeletal pain, making them promising interventions for the treatment of pain in individuals with obesity. Additionally, they are practical and low-cost interventions.

EVOO + DieTBra presented a reduction of over 22% in severe pain, and it was significantly greater compared to EVOO or DieTBra alone. There are no previous reports on this type of intervention in individuals with severe obesity. Clinical trials and cohort studies of dietary interventions with the Mediterranean diet—in which olive oil is a staple food—as well as the use of olive oil extracts rich in polyphenols, have demonstrated pain improvements in individuals with rheumatoid arthritis and osteoarthritis [18,21,49]. A clinical trial evaluating the intake of aqueous extracts of olive leaves showed significant improvements in pain when walking, climbing, or descending stairs and sleeping [50]. Topical use of olive oil has also shown beneficial results in joint pain [20]. Based on our results, nutritional intervention with EVOO + DieTBra is promising for nonpharmacological treatment of severe pain, and can be used in conjunction with other treatments.

Considering pain by body site, DieTBra showed more favorable results in pain reduction than the other interventions. DieTBra promoted significant reduction in wrist and hand pain compared to EVOO + DieTBra, and significant reduction in knee pain compared to EVOO alone. There was a significant reduction in upper back pain for all of the intervention groups, with the greatest reduction in the DieTBra group (approximately 27%, compared to nearly 10% for EVOO and 16% for EVOO + DieTBra). There have been no previous studies that reported the effects of the traditional Brazilian diet on musculoskeletal pain. The few available studies on dietary patterns and pain were performed with the Mediterranean diet [21]. However, other nutritional interventions have been tested on musculoskeletal pain [15,16,50]. For example, strawberry consumption significantly reduced chronic pain in adults with obesity and knee osteoarthritis [51], and a vegan diet improved pain scores in overweight adults with fibromyalgia [15], while gluten-free and low-caloric diets did not significantly reduce pain in individuals with fibromyalgia [16]. The reason that DieTBra was favorable in the reduction of musculoskeletal pain may be due to its characteristics as a healthy, plant-based dietary pattern, especially considering the consumption of rice, beans, fruits, and vegetables [52].

Nutritional interventions may have effects in reducing chronic musculoskeletal pain, reducing comorbidities—such as obesity and cardiovascular diseases—and, thus, reducing health costs [10,11]. It is important to highlight that there is a lack of research on the effectiveness of diet therapy for people with chronic pain, and on the barriers to its implementation in clinical practice. In this sense, we consider our findings to be clinically important. Thus, we encourage future research to evaluate the benefits of nutritional interventions—such as DieTBra and the consumption of olive oil—with different populations in the long term.

The Mediterranean diet is associated with many health benefits; however, it implies great modifications in the food pattern, and it can be difficult to adapt to other countries with a food culture different to that of the Mediterranean. Additionally, in some countries, the accessibility to certain food items of the Mediterranean diet is limited. DieTBra, however, is more accessible and easier to practice, as it is similar to the usual diet of the populations of South America, Central America, and Asia, based on the consumption of rice, beans, seasonal fruits, and vegetables. Thus, it becomes easily incorporated in the treatment of pain in adults in a variety of countries.

One limitation of this study is the lack of a control group without nutritional intervention. However, it is worth noting that in studies with nutritional intervention it is often difficult to establish a comparative group without any intervention (control group). In addition, our research was carried out in partnership with an outpatient reference service for the clinical treatment of people with severe obesity. Leaving these patients with severe obesity and full of comorbidities without any type of treatment is considered unethical by Ethics Committees. It is important to highlight that the initial goal of obesity treatment is usually modest weight loss—5% to 10% of total weight. Appropriate treatment methods depend on the severity of obesity, general health, and willingness to participate in a weight loss plan. Reducing calories and encouraging changes in eating habits are vital to overcoming obesity. Thus, in conceptualizing this study, we decided to use three intervention groups: individualized diet with a prescription based on the traditional Brazilian diet (DieTBra), extra-virgin olive oil, and the two interventions combined. Another limitation was the follow-up time, which could be extended for us to assess the long-term effects of these interventions, but few clinical trials with this type of intervention have a longer duration. The strengths of the study include its randomized design, the team training to conduct research, the methodological care in the implementation of all stages of the study, the low follow-up losses (10.73%), the use of valid objective measures such as accelerometry to evaluate MVPA, and the evaluation of pain in different body regions.

Nutritional interventions in the treatment of pain are scarce; however, as this study shows, they may be an ally in pain management for individuals with obesity. Our findings suggest that the nutritional interventions with DieTBra + EVOO and DieTBra alone are promising in the treatment of pain intensity, and provide clinical benefits in reducing pain in many body sites. On the other hand, EVOO alone was not effective in reducing pain in our study. The treatment with both diet and olive oil seems to present better results, but if it is impossible to include 52 mL of extra-virgin olive oil in the dietary routine, the adoption of a healthy eating pattern, such as the traditional Brazilian diet, can bring satisfactory results on its own. The consumption of DieTBra + EVOO can be an ally in the treatment of pain in adults with obesity in primary health care, with the advantage of being a nonpharmacological intervention, safe, and without side effects.

## 5. Conclusions

In conclusion, this study provides evidence that DieTBra + EVOO and DieTBra are effective interventions to reduce musculoskeletal pain in individuals with obesity and severe obesity, and that these interventions have great potential for clinical application.

## Figures and Tables

**Table 1:** Demographic characteristics, lifestyle, clinical conditions, food consumption, and musculoskeletal pain of the study groups.

	Total *n* = 149 *n* (%)	EVOO *n* = 50 *n* (%)	DieTBra *n* = 49 *n* (%)	EVOO + DieTBra *n* = 50 *n* (%)	*p* *
**Sex**					
Female	127(85.2)	45(35.4)	40(31.5)	42(33.1)	0.480
Male	22(14.8)	5(22.7)	9(40.9)	8(36.4)	
**Age**					
<40	83(55.7)	32(38.6)	26(31.3)	25(30.1)	0.334
≥40	66(44.3)	18(27.3)	23(34.9)	25(37.9)	
**Smoking**					
No	100(67.1)	32(32.0)	36(36.0)	32(32.0)	0.513
Former smoker and smoker	49(32.9)	18(36.7)	13(26.5)	18(36.7)	
**Binge drinking**					
No	106(71.1)	35(33.0)	36(34.0)	35(33.0)	0.908
Yes	43(28.9)	15(34.9)	13(30.2)	15(34.9)	
**MVPA (min/day)**					
<Median (8.39)	71(50.7)	21(29.6)	21(29.6)	29(40.9)	0.180
≥Median (8.39)	69(49.3)	26(37.7)	25(36.2)	18(26.1)	
**Degree of obesity (kg/m^2^)**					
35–39.9	25(16.8)	9(36.0)	8(32.0)	8(32.0)	0.666
40–49.9	84(56.4)	25(29.8)	27(32.1)	32(38.1)	
≥50	40(26.9)	16(40.0)	14(35.0)	10(25.0)	
**Use of analgesics**					
No	84(56.4)	24(28.6)	30(35.7)	30(35.7)	0.339
Yes	65(43.6)	26(40.0)	19(29.2)	20(30.8)	
**Use of anti-inflammatories**					
No	115(77.2)	38(33.0)	39(33.9)	38(33.0)	0.887
Yes	34(22.8)	12(35.3)	10(29.4)	12(35.3)	
**Use of muscle relaxant**					
No	67(45.0)	18(26.9)	26(38.8)	23(34.3)	0.230
Yes	82(55.0)	32(39.0)	23(28.0)	27(32.9)	
**Arthritis/arthrosis**					
No	118(79.2)	36(30.5)	40(33.9)	42(35.6)	0.294
Yes	31(20.8)	14(45.2)	9(29.0)	8(25.8)	
**Depression**					
No	54(36.2)	15(27.8)	19(35.2)	20(37.0)	0.526
Yes	95(63.8)	35(36.8)	30(31.6)	30(31.6)	
**Anxiety**					
No	41(27.5)	12(29.3)	19(46.3)	10(24.4)	0.089
Yes	108(72.5)	38(35.2)	30(27.8)	40(37.0)	
**C-reactive protein (mg/dL)**					
Nonreactive	108(72.5)	34(31.5)	37(34.3)	37(34.3)	0.675
Reagent	41(27.5)	16(39.0)	12(29.3)	13(31.7)	
**Vitamin D (ng/mL)**					
Deficiency	29(19.5)	12(41.4)	16(31.4)	22(31.9)	0.206
Insufficiency	51(34.2)	12(41.4)	13(25.5)	24(34.8)	
Sufficiency	69(46.3)	5(17.2)	22(43.1)	23(33.3)	
**Consumption of fruit (daily)**					
No	109(73.1)	38(34.9)	37(33.9)	34(31.2)	0.600
Yes	40(26.9)	12(30.0)	12(30.0)	16(40.0)	
**Vegetable consumption (daily)**					
No	108(72.5)	38(35.2)	34(31.5)	36(33.3)	0.759
Yes	41(27.5)	12(29.3)	15(36.6)	14(34.1)	
**Pain**					
No	16(10.7)	5(31.3)	5(31.3)	6(37.50)	0.939
Yes	133(89.3)	45(33.8)	44(33.1)	44(33.1)	
**Severe pain**					
No	46(30.9)	17(37.0)	18(39.1)	11(23.9)	0.239
Yes	103(69.1)	33(32.0)	31(30.1)	39(37.9)	
**Multisite pain**					
0	16(10.7)	5(31.3)	5(31.3)	6(37.5)	0.946
1–3	53(35.6)	20(37.7)	15(28.3)	18(34.0)	
4–5	40(26.9)	13(32.5)	13(32.5)	14(35.0)	
6–9	40(26.9)	12(30.0)	16(40.0)	12(30.0)	
**Pain by body regions**					
**Wrists and hands**					
No	83(55.7)	28(33.7)	25(30.1)	30(36.1)	0.667
Yes	66(44.3)	22(33.3)	24(36.4)	20(30.3)	
**Upper back**					
No	71(47.7)	28(39.4)	21(29.6)	22(31.0)	0.347
Yes	78(52.4)	22(28.2)	28(35.9)	28(35.9)	
**Lower back**					
No	56(37.6)	22(39.3)	14(25.0)	20(35.7)	0.259
Yes	93(62.4)	28(30.1)	35(37.6)	30(32.3)	
**Hip**					
No	97(65.1)	34(35.1)	33(34.0)	30(30.9)	0.648
Yes	52(34.9)	16(30.8)	16(30.8)	20(38.5)	
**Knees**					
No	70(47.0)	25(35.7)	24(34.3)	21(30.0)	0.684
Yes	79(53.0)	25(31.7)	25(31.7)	29(36.7)	
**Ankles and feet**					
No	46(30.87)	15(32.6)	16(34.8)	15(32.6)	0.947
Yes	103(69.1)	35(34.0)	33(32.0)	35(34.0)	

*: Pearson’s chi-squared test; EVOO: extra-virgin olive oil group; DieTBra: traditional Brazilian diet group; EVOO + DieTBra: traditional Brazilian diet plus extra-virgin olive oil group; MVPA: moderate-to-vigorous-intensity physical activity. Item titles are in bold in the table. Original to this manuscript.

**Table 2:** Comparison of the prevalence of pain and intensity of musculoskeletal pain at baseline and at the end of the study for each intervention group (*n* = 149).

	EVOO			DieTBra			EVOO + DieTBra		
	Baseline (*n* = 50) *n* (%)	Final Follow-Up (*n* = 43) *n* (%)	*p* *	Baseline (*n* = 49) *n* (%)	Final Follow-Up (*n* = 43) *n* (%)	*p* *	Baseline (*n* = 50) *n* (%)	Final Follow-Up (*n* = 47) *n* (%)	*p* *
Pain (yes%)	45 (90.0)	37 (86.0)	0.480	44 (89.8)	40 (93.0)	0.157	44 (88.0)	40 (85.1)	0.655
Pain intensity—Severe pain (yes%)	33 (66.0)	25 (58.1)	0.405	31 (63.3)	23 (53.5)	0.197	39 (78.0)	26 (55.3)	**0.002**
Pain by body site (%)									
Wrists and hands	22 (44.0)	19 (44.2)	0.527	24 (49.0)	15 (34.9)	0.058	20 (40.0)	13 (27.7)	0.109
Upper back	22 (44.0)	15 (34.9)	0.134	28 (57.1)	13 (30.2)	**0.001**	28 (56.0)	19 (40.4)	0.180
Lower back	28 (56.0)	23 (53.5)	0.564	35 (71.4)	29 (67.4)	0.527	30 (60.0)	24 (51.1)	0.366
Hip	16 (32.0)	9 (20.9)	0.132	16 (32.5)	11 (25.6)	0.317	20 (40.0)	15 (31.9)	0.368
Knees	25 (50.0)	19 (44.2)	0.285	25 (51.0)	14 (32.6)	**0.046**	29 (58.0)	22 (46.8)	0.166
Ankles and feet	35 (70.0)	28 (65.1)	0.564	33 (37.3)	58 (65.1)	0.317	35 (70.0)	29 (61.7)	0.248

EVOO: extra-virgin olive oil group; DieTBra: traditional Brazilian diet group; EVOO + DieTBra: extra-virgin olive oil + DieTBra; *: McNemar’s test. Bold format means significant difference. Original to this manuscript.

**Table 3:** Comparison of percentage change in the prevalence of the outcome variables according to intervention group, after 12-week follow-up.

Difference	EVOO	DieTBra	EVOO + DieTBra	EVOO × DieTBra *	EVOO × EVOO + DieTBra *	DieTBra × EVOO + DieTBra *
				*p*	*p*	*p*
Δ Pain (yes%)	**−4**	**+3.2**	−2.9	1.000	0.405	0.705
Δ Pain intensity—Severe pain (yes%)	−7.9	−9.8	−22.7	0.131	**0.007**	**0.003**
Δ Pain by body site (yes%)						
Wrists and hands	+0.2	−14.1	−12.3	0.074	0.102	**0.014**
Upper back	−9.1	−26.9	−15.6	**0.001**	**0.045**	**0.002**
Lower back	−2.5	−4	−8.9	0.394	0.297	0.275
Hip	−11.1	−6.9	−8.1	0.074	0.088	0.179
Knees	−5.8	−18.4	−11.2	**0.028**	0.083	**0.016**
Ankles and feet	−4.9	+27.8	−8.3	0.275	0.221	0.126

EVOO: extra-virgin olive oil group; DieTBra: traditional Brazilian diet group; EVOO + DieTBra: extra-virgin olive oil + DieTBra; *: Pearson’s chi-squared test; Δ: percentage difference between baseline and final follow-up. Bold format means significant difference. Original to this manuscript.

## Data Availability

The data presented in this study are available on request from the corresponding author.

## Supplementary Materials

The following are available online at https://www.mdpi.com/article/10.3390/ijerph182111112/s1, Table S1: Comparison of the prevalence of pain, intensity of musculoskeletal pain and pain by body site after the 12-week follow-up according to intervention group.

## References

[1] Afshin A., Forouzanfar M.H., Reitsma M.B., Sur P., Estep K., Lee A., Marczak L., Mokdad A.H., Moradi-Lakeh M., Naghavi M. (2017). Health Effects of Overweight and Obesity in 195 Countries over 25 Years. N. Engl. J. Med..

[2] Vincent H.K., Adams M.C., Vincent K.R., Hurley R.W. (2013). Musculoskeletal pain, fear avoidance behaviors, and functional decline in obesity: Potential interventions to manage pain and maintain function. Reg. Anesth. Pain Med..

[3] Calenzani G., Santos F.F.D., Wittmer V.L., Freitas G.K.F., Paro F.M. (2017). Prevalence of musculoskeletal symptoms in obese patients candidates for bariatric surgery and its impact on health related quality of life. Arch. Endocrinol. Metab..

[4] MacLellan G.A., Dunlevy C., O’Malley E., Blake C., Breen C., Gaynor K., Wallace N., Yoder R., Casey D., Mehegan J. (2017). Musculoskeletal pain profile of obese individuals attending a multidisciplinary weight management service. Pain.

[5] Magnusson K., Østerås N., Mowinckel P., Natvig B., Hagen K. (2014). No strong temporal relationship between obesity and multisite pain–results from a population-based 20-year follow-up study. Eur. J. Pain.

[6] Mundal I., Bjørngaard J.H., Nilsen T.I., Nicholl B.I., Gråwe R.W., Fors E.A. (2016). Long-term changes in musculoskeletal pain sites in the general population: The HUNT study. J. Pain.

[7] Perrot S., Cohen M., Barke A., Korwisi B., Rief W., Treede R.-D. (2019). The ITftCoCP: The IASP classification of chronic pain for ICD-11: Chronic secondary musculoskeletal pain. Pain.

[8] Coggon D., Ntani G., Palmer K.T., Felli V.E., Harari R., Barrero L.H., Felknor S.A., Gimeno D., Cattrell A., Vargas-Prada S. (2013). Patterns of multisite pain and associations with risk factors. Pain.

[9] Rondanelli M., Faliva M.A., Miccono A., Naso M., Nichetti M., Riva A., Guerriero F., De Gregori M., Peroni G., Perna S. (2018). Food pyramid for subjects with chronic pain: Foods and dietary constituents as anti-inflammatory and antioxidant agents. Nutr. Res. Rev..

[10] Mendonça C.R., Noll M., Castro M.C.R., Silveira E.A. (2020). Effects of Nutritional Interventions in the Control of Musculoskeletal Pain: An Integrative Review. Nutrients.

[11] Elma Ö., Yilmaz S.T., Deliens T., Coppieters I., Clarys P., Nijs J., Malfliet A. (2020). Do Nutritional Factors Interact with Chronic Musculoskeletal Pain? A Systematic Review. J. Clin. Med..

[12] Carrijo A.P., Botelho R.B.A., Akutsu R., Zandonadi R.P. (2018). Is What Low-Income Brazilians Are Eating in Popular Restaurants Contributing to Promote Their Health?. Nutrients.

[13] Tedeschi S.K., Frits M., Cui J., Zhang Z.Z., Mahmoud T., Iannaccone C., Lin T.C., Yoshida K., Weinblatt M.E., Shadick N.A. (2017). Diet and Rheumatoid Arthritis Symptoms: Survey Results From a Rheumatoid Arthritis Registry. Arthritis Care Res..

[14] Dyer J., Davison G., Marcora S.M., Mauger A.R. (2017). Effect of a Mediterranean Type Diet on Inflammatory and Cartilage Degradation Biomarkers in Patients with Osteoarthritis. J. Nutr. Health Aging.

[15] Kaartinen K., Lammi K., Hypen M., Nenonen M., Hänninen O., Rauma A.-L. (2000). Vegan diet alleviates fibromyalgia symptoms. Scand. J. Rheumatol..

[16] Slim M., Calandre E.P., Garcia-Leiva J.M., Rico-Villademoros F., Molina-Barea R., Rodriguez-Lopez C.M., Morillas-Arques P. (2017). The Effects of a gluten-free diet versus a hypocaloric diet among patients with fibromyalgia experiencing gluten sensitivity–like symptoms. J. Clin. Gastroenterol..

[17] Cardeno A., Sanchez-Hidalgo M., Alarcon-de-la-Lastra C. (2013). An up-date of olive oil phenols in inflammation and cancer: Molecular mechanisms and clinical implications. Curr. Med. Chem..

[18] Bitler C.M., Matt K., Irving M., Hook G., Yusen J., Eagar F., Kirschner K., Walker B., Crea R. (2007). Olive extract supplement decreases pain and improves daily activities in adults with osteoarthritis and decreases plasma homocysteine in those with rheumatoid arthritis. Nutr. Res..

[19] Gelmini F., Ruscica M., Macchi C., Bianchi V., Maffei Facino R., Beretta G., Magni P. (2016). Unsaponifiable Fraction of Unripe Fruits of Olea europaea: An Interesting Source of Anti-inflammatory Constituents. Planta Med..

[20] Nakhostin-Roohi B., Khoshkhahesh F., Bohlooli S. (2016). Effect of virgin olive oil versus piroxicam phonophoresis on exercise-induced anterior knee pain. Avicenna J. Phytomed..

[21] Veronese N., Koyanagi A., Stubbs B., Cooper C., Guglielmi G., Rizzoli R., Punzi L., Rogoli D., Caruso M.G., Rotolo O. (2019). Mediterranean diet and knee osteoarthritis outcomes: A longitudinal cohort study. Clin. Nutr..

[22] Cardoso C.K.S., Santos A., Rosa L.P.S., Mendonça C.R., Vitorino P.V.O., Peixoto M., Silveira E.A. (2020). Effect of Extra Virgin Olive Oil and Traditional Brazilian Diet on the Bone Health Parameters of Severely Obese Adults: A Randomized Controlled Trial. Nutrients.

[23] Rodrigues A., Rosa L.P.S., da Silva H.D., Silveira-Lacerda E.P., Silveira E.A. (2018). The Single Nucleotide Polymorphism PPARG2 Pro12Ala Affects Body Mass Index, Fat Mass, and Blood Pressure in Severely Obese Patients. J. Obes..

[24] Rodrigues A.P.S., Rosa L.P.S., Silveira E.A. (2018). PPARG2 Pro12Ala polymorphism influences body composition changes in severely obese patients consuming extra virgin olive oil: A randomized clinical trial. Nutr. Metab..

[25] Santos A., Rodrigues A., Rosa L.P.S., Noll M., Silveira E.A. (2020). Traditional Brazilian Diet and Olive Oil Reduce Cardiometabolic Risk Factors in Severely Obese Individuals: A Randomized Trial. Nutrients.

[26] Santos A.S., Rodrigues A.P.S., Rosa L.P., Sarrafzadegan N., Silveira E.A. (2020). Cardiometabolic risk factors and Framingham Risk Score in severely obese patients: Baseline data from DieTBra trial. Nutr. Metab. Cardiovasc. Dis..

[27] Wadden T.A., Webb V.L., Moran C.H., Bailer B.A. (2012). Lifestyle modification for obesity: New developments in diet, physical activity, and behavior therapy. Circulation.

[28] Monteiro C.A., Cannon G. (2012). The impact of transnational “big food” companies on the South: A view from Brazil. PLoS Med..

[29] De Atenção Básica D., de Atenção à Saúde S., da Saúde B.M. (2014). Guia Alimentar para a População Brasileira/Ministério da Saúde, Secretaria de Atenção à Saúde, Departamento de Atenção Básica.

[30] Hall K.D. (2008). What is the required energy deficit per unit weight loss?. Int. J. Obes..

[31] Horie L.M., Gonzalez M.C., Torrinhas R.S., Cecconello I., Waitzberg D.L. (2011). New specific equation to estimate resting energy expenditure in severely obese patients. Obesity.

[32] Hill J.O., Wyatt H.R., Peters J.C. (2012). Energy balance and obesity. Circulation.

[33] Trumbo P., Schlicker S., Yates A.A., Poos M. (2002). Dietary reference intakes for energy, carbohdrate, fiber, fat, fatty acids, cholesterol, protein and amino acids. J. Acad. Nutr. Diet..

[34] Satija A., Yu E., Willett W.C., Hu F.B. (2015). Understanding nutritional epidemiology and its role in policy. Adv. Nutr..

[35] Schulze M.B., Martínez-González M.A., Fung T.T., Lichtenstein A.H., Forouhi N.G. (2018). Food based dietary patterns and chronic disease prevention. BMJ.

[36] McCormack V.A., Agudo A., Dahm C.C., Overvad K., Olsen A., Tjonnelan A., Kaaks R., Boeing H., Manjer J., Almquist M. (2010). Cigar and pipe smoking and cancer risk in the European Prospective Investigation into Cancer and Nutrition (EPIC). Int. J. Cancer.

[37] Bloomfield K., Gmel G., Wilsnack S. (2006). Introduction to special issue ‘Gender, Culture and Alcohol Problems: A Multi-national Study’. Alcohol. Suppl..

[38] Kanny D., Naimi T.S., Liu Y., Lu H., Brewer R.D. (2018). Annual total binge drinks consumed by US adults, 2015. Am. J. Prev. Med..

[39] Lohman T.G., Roche A.F., Martorell R. (1988). Anthropometric Standardization Reference Manual.

[40] World Health Organization (2000). Obesity: Preventing and Managing the Global Epidemic.

[41] Zigmond A., Snaith R. (1983). The hospital anxiety and depression scale *Acta Psychiatr*. Scand..

[42] World Health Organization (2018). WHO Collaborating Centre for Drug Statistics Methodology.

[43] Holick M.F., Binkley N.C., Bischoff-Ferrari H.A., Gordon C.M., Hanley D.A., Heaney R.P., Murad M.H., Weaver C.M. (2011). Evaluation, treatment, and prevention of vitamin D deficiency: An Endocrine Society clinical practice guideline. J. Clin. Endocrinol. Metab..

[44] Thompson F., Subar A. (2008). Dietary Assessment Methodology. Nutrition in the Prevention and Treatment of Disease.

[45] De Barros E.N., Alexandre N.M. (2003). Cross-cultural adaptation of the Nordic musculoskeletal questionnaire. Int. Nurs. Rev..

[46] Mendonça C.R., Noll M., Silveira E.A. (2018). Adaptation and validation of body maps for musculoskeletal pain location in patients with severe obesity. Korean J. Pain.

[47] Kuorinka I., Jonsson B., Kilbom A., Vinterberg H., Biering-Sørensen F., Andersson G., Jørgensen K. (1987). Standardised Nordic questionnaires for the analysis of musculoskeletal symptoms. Appl. Ergon..

[48] Boonstra A.M., Stewart R.E., Koke A.J., Oosterwijk R.F., Swaan J.L., Schreurs K.M., Schiphorst Preuper H.R. (2016). Cut-Off Points for Mild, Moderate, and Severe Pain on the Numeric Rating Scale for Pain in Patients with Chronic Musculoskeletal Pain: Variability and Influence of Sex and Catastrophizing. Front. Psychol..

[49] McKellar G., Morrison E., McEntegart A., Hampson R., Tierney A., Mackle G., Scoular J., Scott J.A., Capell H.A. (2007). A pilot study of a Mediterranean-type diet intervention in female patients with rheumatoid arthritis living in areas of social deprivation in Glasgow. Ann. Rheum. Dis..

[50] Takeda R., Koike T., Taniguchi I., Tanaka K. (2013). Double-blind placebo-controlled trial of hydroxytyrosol of Olea europaea on pain in gonarthrosis. Phytomedicine.

[51] Schell J., Scofield R.H., Barrett J.R., Kurien B.T., Betts N., Lyons T.J., Zhao Y.D., Basu A. (2017). Strawberries improve pain and inflammation in obese adults with radiographic evidence of knee osteoarthritis. Nutrients.

[52] Nascimento S., Barbosa F.S., Sichieri R., Pereira R.A. (2011). Dietary availability patterns of the brazilian macro-regions. Nutr. J..

