# Supplementary material for: Reduction in Pain and Pain Intensity with Nonpharmacological Treatment in Severely Obese Patients: A Randomized Clinical Trial
Source: Int J Environ Res Public Health. 2021 Oct 22;18(21):11112. doi: 10.3390/ijerph182111112 (PMC8582684; doi:10.3390/ijerph182111112)
Supplement: Supplementary file 1 [file ijerph-1416832-supplementary.pdf › ijerph-1416832-supplementary.pdf]

**Supplementary Table S1.** Comparison of the prevalence of pain, intensity of musculoskeletal pain and pain by body site after the 12-week follow-up according to intervention group.

| | EVOO (n=43) | DieTBra (n=43) | EVOO + DieTBra (n=47) | All | EVOO x DieTBra | EVOO x EVOO + DieTBra | DieTBra x EVOO + DieTBra |
|---|---|---|---|---|---|---|---|
| | n (%) | n (%) | n (%) | P* | P* | P* | P* |
| Pain (yes%) | 37 | 40 (93.0) | 40 (85.1) | 0.47 | 0.483 | 0.899 | 0.320 |
| Intense pain (yes%) | 25 | 23 (53.5) | 26 (55.3) | 0.90 | 0.664 | 0.787 | 0.862 |
| Pain by body site (%) | | | | | | | |
| Wrist and hand | 19 | 15 (34.9) | 13 (27.7) | 0.26 | 0.378 | 0.102 | 0.460 |
| Upper back | 15 | 13 (30.2) | 19 (40.4) | 0.59 | 0.645 | 0.588 | 0.313 |
| Lower back | 23 | 29 (67.4) | 24 (51.1) | 0.24 | 0.186 | 0.818 | 0.115 |
| Hip | 9 (20.9) | 11 (25.6) | 15 (31.9) | 0.49 | 0.610 | 0.239 | 0.508 |
| Knees | 19 | 14 (32.6) | 22 (46.8) | 0.35 | 0.268 | 0.803 | 0.168 |
| Ankles and feet | 28 | 58 (65.1) | 29 (61.7) | 0.92 | 1.000 | 0.737 | 0.737 |

* Pearson's chi-squared test; EVOO, extra virgin olive oil group; DieTBra, traditional Brazilian diet group; Extra virgin olive oil + DieTBra (EVOO + DieTBra).
